# Husband’s involvement in maternal antenatal care and associated factors among pregnant women’s: study protocol for systematic review and meta-analysis

**DOI:** 10.1016/j.xagr.2025.100532

**Published:** 2025-06-06

**Authors:** Aster Shiferaw, Mulunesh Minale

**Affiliations:** Debre Markos University, College of Health Science Department of Midwifery, Debre Markos, Ethiopia

**Keywords:** associated factors, husbands’ involvement, maternal ANC, protocol, systematic review and meta-analysis

## Abstract

**Background:**

The evidence of husband’s involvement in maternal antenatal care (ANC) is scant and inconsistence across each study. Therefore this systematic review and meta-analysis will be to examine (1) the characteristics of published studies on husband involvement in maternal ANC; (2) estimate the nationwide magnitude of husband involvement in maternal ANC;(3) show the significant association between husband involvement in maternal ANC and reported associated factors in published articles.

**Method:**

This systematic review and meta-analysis will be conducted by publishing and unpublishing articles, which will be searched online through Google Scholar, PubMed, Cochrane Library, EMBASE, HINARI, and Africa Journals. The Preferred Reporting Item for Systematic Review and Meta-Analysis (PRISMA) guideline will be used to review articles. Repeated articles will be removed by EndNote version X7.1. The data will be extracted by using the 2014 Joanna Briggs Institute Reviewers’ Manual data extraction. Critical appraisal of the studies will be done by the Joanna Briggs Institute Meta-Analysis of Statistics Assessment and Review Instrument (JBI-MAStARI) for cross-sectional studies tool. Begg-Mazumdar rank correlation tests and Egger’s regression tests will be used to check the presence of publication bias. The presence of heterogeneity will be assessed by Cochran’s Q statistics.

**Discussion:**

This systematic review and meta-analysis will be the only study that investigates the magnitude and associated factors of husband’s involvement in maternal ANC. Through gathering information, the possible outcome will be to investigate and show those interested to utilize it.

**AJOG Global Reports at a Glance**

**Why was this study conducted?**

Publish of this article will help the researchers to have insight about the topic introduction and can be reference for those who desire to conduct study on this title.

**Key findings?**

Initiates the researcher to conduct research if it is not addressed in their residence.

**What does this add to what is known?**

It can be used as input for those interested to conduct systematic review and meta-analysis and show the nationwide figure of the title.

## Background

Pregnant women and adolescents can get comprehensive reproductive health services through the antenatal care (ANC) as a platform. Thus, by supplying high-quality ANC, the ANC not only ensures the health of the mother and the unborn child but also helps a woman and her family have a happy and healthy pregnant experience.^1^ By 2030, the goal of Sustainable Development Goal target 3.1 is to lower maternal mortality to fewer than 70 deaths per 100,000 live births. However, in 2020, almost 800 women per day passed away from pregnancy- and childbirth-related avoidable causes; that is, one woman passes away every 2 minutes.^2^

In low- and middle-income nations, nearly all maternal deaths (99%) and child deaths (98%) took place. If the teenage girls or pregnant women had had access to high-quality ANC, these maternal deaths might have been avoided.^3^ The main tactic used by the government is prenatal care (ANC), which aims to lower mother morbidity and mortality. A healthy lifestyle is encouraged, all pregnancy issues are detected, appropriate action is taken, complaints are addressed, labor and delivery are prepared for, and an early approach to monitoring and maintaining the health and safety of mothers and fetuses is called ANC. Early detection and treatment of any unanticipated problems during pregnancy are critical components of prenatal care.^4^ In patriarchal environments, males have a great deal of influence over their spouses, and a woman needs her husband’s approval and financial support before she can seek medical attention.^5^ It is important to remember that most societies in sub-Saharan Africa, including Ethiopia, are patriarchal, and most males tend to view pregnancy and childbirth as tasks that belong to women.^6^ The societal norm for a man is to possess strength. Participating in maternal health initiatives could be viewed as stereotypically feminine and interpreted as a show of weakness or submission to authority from women. This could result in social stigma, which would discourage additional guys from participating.^7^ There is great significance for bettering community health, fostering stronger family ties, and increasing mother and child health outcomes in Africa if more men participate in maternal healthcare. Men are discouraged from engaging despite the benefits because of barriers related to culture, social status, and the healthcare system.^7^ Men helping with maternal care guarantees that women will believe their male spouses when they ask for Consistent attendance at prenatal visits, Following the advice of medical professionals and paying attention to prompt interventions.^7^ But studies shows that only a little over half of male partners accompanied their wife in ANC.^8^ Several studies conducted in various countries showed that one of the ANC barriers was poor husbands’ support and involvement in the antenatal and labor processes. The poor of husbands’ permission is why women delay maternal health care.^9^ Even though studies conducted on husband’s involvement in ANC, study that represents the nationwide figure of husband’s involvement in ANC is important. Therefore this study will show the result that represents the country level of husband’s involvement in ANC. The result of this study will be used as input for responsible body to take appropriate measures.

## Objective

The aim of this systematic review and meta-analysis will be to examine (1) the characteristics of published studies on husband involvement in maternal ANC and (2) estimate the nationwide magnitude of husband involvement in maternal ANC; (3) show the significant association between husband involvement in maternal ANC and reported associated factors in published articles.

## Methods

### Study design and searching strategies

This systematic review and meta-analysis will be conducted by using published and unpublished articles on husbands’ involvement in maternal ANC and associated factors among pregnant women in Ethiopia. The articles will be searched online through Google, Google Scholar, PubMed/MEDLINE, Cochrane Library, EMBASE, HINARI, and Africa Journals. Again, the reference list in already searched articles will be examined and included in the study. Those searched articles will be systematically reviewed and analyzed to determine the magnitude of husbands’ participation in maternal ANC and associated factors. The keywords used to search articles will be husband, involvement, maternal ANC, associated factors, and pregnant women. Preferred Reporting Items for Systematic Reviews and Meta-Analyses (PRISMA) guidelines were used to review articles systematically.^10^

### Search strategy developed from PubMed database

Search: (((((husband [MeSH Terms]) OR (spouse [MeSH Terms])) AND (involvement [MeSH Terms])) AND (maternal [MeSH Terms])) OR (wife [MeSH Terms])) AND (antenatal care [MeSH Terms])

(((((husband [MeSH Terms]) OR (spouse [MeSH Terms])) AND (involvement [MeSH Terms])) AND (maternal [MeSH Terms])) OR (wife [MeSH Terms])) AND (antenatal care [MeSH Terms])

Translations

Husband [MeSH Terms]: “spouses”[MeSH Terms]

Spouse [MeSH Terms]: “spouses”[MeSH Terms]

Maternal [MeSH Terms]: “mothers”[MeSH Terms]

Wife [MeSH Terms]: “spouses”[MeSH Terms]

Antenatal care [MeSH Terms]: “prenatal care”[MeSH Terms]

Warnings

(((((husband [MeSH Terms]) OR (spouse [MeSH Terms])) AND (involvement [MeSH Terms])) AND (maternal [MeSH Terms])) OR (wife [MeSH Terms])) AND (antenatal care [MeSH Terms])

### Inclusion criteria

All observational studies that show quantitative findings on the magnitude and association between exposure and related factors conducted on husbands’ involvement in maternal ANC and associated factors among pregnant women will be included. Qualitative studies and others that do not provide enough evidence on the outcome of interest will be excluded.

### Data extraction

The searched articles will be collected, and repeated articles will be removed by using EndNote version X7.1. The data will be extracted by using the 2014 Joanna Briggs Institute Reviewers’ Manual data extraction form, which includes (1) title, author, year of study, year of publication, study design, sample size, study participants, study area, response rate, and sampling method; (2) the magnitude of husbands’ involvement in maternal ANC among pregnant women; and (3) the factor that is associated with husbands’ involvement in maternal ANC.

### Outcome measurement

This systematic review and meta-analysis will have two outcomes: the first one is the magnitude of husbands’ involvement in maternal ANC among pregnant women, and the second one is associated factors of husbands’ involvement in maternal ANC repeatedly reported in at least two previous studies.

### Quality assessment and data collection

Critical appraisal of the studies will be done based on the Joanna Briggs Institute Meta-Analysis of Statistics Assessment and Review Instrument (JBI-MAStARI) for cross-sectional studies tool. Each article will be reviewed independently and reassessed again in another day to solve the inconsistency between the reviews. All articles will have their quality checked before being included in the final review. The articles that have a score of 50% and above will pass for the final systematic review and meta-analysis.^11^

### Data analysis

The extracted data will be entered into a Microsoft Excel spreadsheet and exported to STATA version 17 for analysis. The presence of heterogeneity will be assessed by Cochran’s Q statistic in the random effects model and indicated with inverse variance (I²); the result interprets 25%, 50%, and 75% as low, medium, and high heterogeneity, respectively, at a *P* value of less than .05.^12^ Again, subgroup analysis will be carried out to identify the source of heterogeneity. Begg-Mazumdar rank correlation tests and Egger’s regression tests at *P* values less than .05 will be used to check the presence of publication bias. The estimated magnitude of husbands’ involvement in maternal ANC among pregnant women will be presented by forest plot with its corresponding 95% CI, and the odds ratio will be used to determine the presence of significant association between factors and husbands’ involvement in maternal ANC.^13^

## Operational definition

### Husband involvement in maternal ANC

This refers to couples attending ANC together at the healthcare facility, obtaining information from the healthcare provider, making joint decisions about their actions and destinations, and offering both financial and physical support throughout the pregnancy. The level of male involvement was quantitatively assessed through a composite measure consisting of 6 items, each equally weighted and featuring a dichotomized component (yes/no). Each variable was assigned a score of “1” if it was performed and “0” if it was not, leading to a total score for each participant; a low level of involvement was designated for those scoring ≤ 2, while a high level of involvement was for those scoring ≥3.

### Ethics and dissemination

Ethical approval is not applicable for this study because the data will be collected from published articles. The findings of this study will be accessible to everyone by publishing in a qualified peer-reviewed open-access journal. Again, effort will go into presenting the findings in research conferences and dissertations.

## Discussion

This systematic review and meta-analysis will assess the magnitude of husbands’ involvement in maternal ANC and the factors that are associated with husbands’ involvement in maternal ANC. This is the only study that shows the nationwide figure of husbands’ involvement in maternal ANC and will provide new knowledge regarding the significant relation with determinants. The finding will help to have an understanding of how much husband involvement affects the maternal health care utilization and provides direction to take appropriate measures.

## CRediT authorship contribution statement

**Aster Shiferaw:** Writing – review & editing, Writing – original draft, Conceptualization. **Mulunesh Minale:** Writing – review & editing, Writing – original draft, Conceptualization.
